# Predictive value of neutrophil to lymphocyte ratio for ischemic stroke in patients with atrial fibrillation: A meta-analysis

**DOI:** 10.3389/fneur.2022.1029010

**Published:** 2022-12-12

**Authors:** Ming Lu, Yeying Zhang, Rui Liu, Xiaoming He, Bonan Hou

**Affiliations:** ^1^Internal Medicine-Cardiovascular Department, Xixi Hospital of Hangzhou, Hangzhou, Zhejiang, China; ^2^Department of Anesthesiology, Affiliated Hospital of Hangzhou Normal University, Hangzhou, Zhejiang, China; ^3^Traditional Chinese Medicine Department, Wenxin Street Health Service Center, Hangzhou, Zhejiang, China; ^4^Endocrine Department, The Second Affiliated Hospital of Zhejiang Chinese Medical University, Hangzhou, Zhejiang, China; ^5^Department of Neurology, The Second Affiliated Hospital of Zhejiang Chinese Medical University, Hangzhou, Zhejiang, China

**Keywords:** neutrophil to lymphocyte ratio, ischemic stroke, atrial fibrillation, meta-analysis, review

## Abstract

**Objective:**

Atrial fibrillation (AF) is an important risk factor for stroke, but the currently used CHA2DS2-VASc score has significant limitations in predicting the risk of stroke. It is important to find new biomarkers to predict stroke risk in patients with AF or as a complement to the CHA2DS2-VASc score. Neutrophil-to-lymphocyte ratio (NLR) may be of potential value. This systematic review and meta-analysis evaluated the association between NLR and stroke risk.

**Methods:**

We searched in electronic databases such as PubMed and EMBASE. The final included studies were analyzed by Stata 12.0 software. Subgroup analyses were used to explore sources of heterogeneity. Publication bias was assessed by Egger's test and Begg's test. Sensitivity analyses assessed the stability of outcomes.

**Results:**

A total of 11 studies with a total of 35,221 patients were included. NLR levels are associated with stroke risk in patients with atrial fibrillation (WMD = 0.72, 95%CI = 0.43–1.01). There was a correlation between the occurrence of stroke and NLR level in AF patients (WMD = 1.96, 95%CI = 1.38–2.53). The incidence of stroke was significantly higher in patients with atrial fibrillation with NLR ≥3 than in those with NLR <3 (RR = 1.4, 95%CI = 1.24–1.58).

**Conclusion:**

This study shows that high NLR values are associated with a higher risk of stroke in AF patients. The incidence of stroke in AF patients with NLR ≥3 was 1.4 times higher than that with NLR <3 (*p* < 0.001). NLR may be considered as a complementary risk assessment for CHA2DS2-VASc score, especially for AF patients with CHA2DS2-VASc score <2. NLR may be a potential biomarker for predicting stroke risk in patients with AF.

## Introduction

Atrial fibrillation (AF) is the most common cardiac arrhythmia and is associated with an increased risk of ischemic stroke. In fact, the actual incidence of AF may be higher than reported because a relatively high proportion of AF is subclinical and undiagnosed (1). Even though anticoagulants are currently effective interventions, 22–36% of patients with AF experience ischemic stroke (2, 3). Clinically, the CHA2DS2-VASc score is commonly used to assess stroke risk in patients with AF. The study showed that the sensitivity of CHA2DS2-VASc ≥1 and ≥2 are both 100%, but the specificity is only 7 and 14%, respectively (4). In addition, a meta-analysis of patients with atrial fibrillation with a mean follow-up of 2.2 years found that ischemic stroke occurred in 2.3% of patients with direct oral anticoagulants (5). Of these patients, one-third had a CHA2DS2-VASc score of 0–1. This suggests a limitation of CHA2DS2-VASc for low-score patients. Therefore, there is a clinical need for new markers that are easy to measure and reliable to improve the assessment of stroke risk after AF or as a complement to the CHA2DS2-VASc score.

The ratio of neutrophils to lymphocytes (NLR) has been a research hotspot in recent years. Because inflammation is an important pathological process in stroke, which is directly related to the prognosis of patients, and NLR is one of the important reference indicators of inflammation (6). NLR is also an important predictor of AF prognosis (7). It has been reported that NLR can be used to assess stroke risk and prognosis in AF patients (8–10), but there are also studies that suggest that NLR is not an independent biomarker for predicting stroke (11). Therefore, the ability of NLR to independently assess or predict stroke risk in AF patients remains unclear. The aim of this systematic review and meta-analysis was to elucidate the association of NLR with stroke risk in patients with AF and its value as a complementary tool to the CHA2DS2-VASc score in assessing stroke risk after AF.

## Methods

### Study selection

Studies were included if they met the following criteria: (1) Study subjects were patients with AF; (2) Articles include randomized controlled trials (RCTs), cohort, case-control studies and cross-sectional studies; (3) involving human subjects; (4) The data tested included NLR; (5) There was a history of ischemic stroke or stroke risk assessment; (6) Written in English or translated into English, as well as Chinese.

Exclusion was non-peer-reviewed manuscripts, conference abstracts, and gray literature.

### Search strategy

The retrieval period is from the date of establishment of the database to June 6, 2022. Databases searched include: PubMed, Cochrane, EMBASE and major Chinese databases (CAJD, CBMdisc, CDFD, CMFD). Searches include “neutrophil to lymphocyte ratio,” “neutrophil to lymphocyte ratio,” “neutrophil/lymphocyte ratio,” “NLR,” “lymphocytes,” “atrial fibrillation,” “AF,” “cerebral infarction” or “stroke,” the search strategy is a combination of the above keywords. In addition, omissions were prevented by reviewing the references of the included studies and the included literature of the relevant meta-analyses.

### Data extraction

Two investigators (ZYY, LR) independently screened articles and resolved disagreements with the intervention of a third investigator (HBN). Two authors (HBN, LM) independently extracted data on general study information (author, year of publication, country), baseline demographics, and clinical characteristics (age, sex, NLR, stroke history, stroke risk score). A third author (HXM) verified all extracted data. Any disagreements were resolved by the three authors through face-to-face discussions.

### Quality assessment

AS the inclusions were case-control studies, three researchers (LM, ZYY, and LR) independently assessed study quality through the Newcastle-Ottawa scale. Overall research quality scores range from low to high on a scale of 0 to 9.

### Statistical analysis

Statistical analysis was performed using Stata 11.0 software. The pooled effect for continuous data is the weighted mean difference (WMD), and the pooled effect for dichotomous data is the hazard ratio with a 95% confidence interval. Depending on heterogeneity, a random-effects or fixed-effects model was chosen. In random-effects models, *I*^2^ ≥ 50% or *p* ≤ 0.10 indicated significant heterogeneity, and we performed subgroup analyses to identify possible sources. The possibility of publication bias was assessed using the Begg and Egger test.

## Results

### Characteristics of included studies

**Figure 1:**
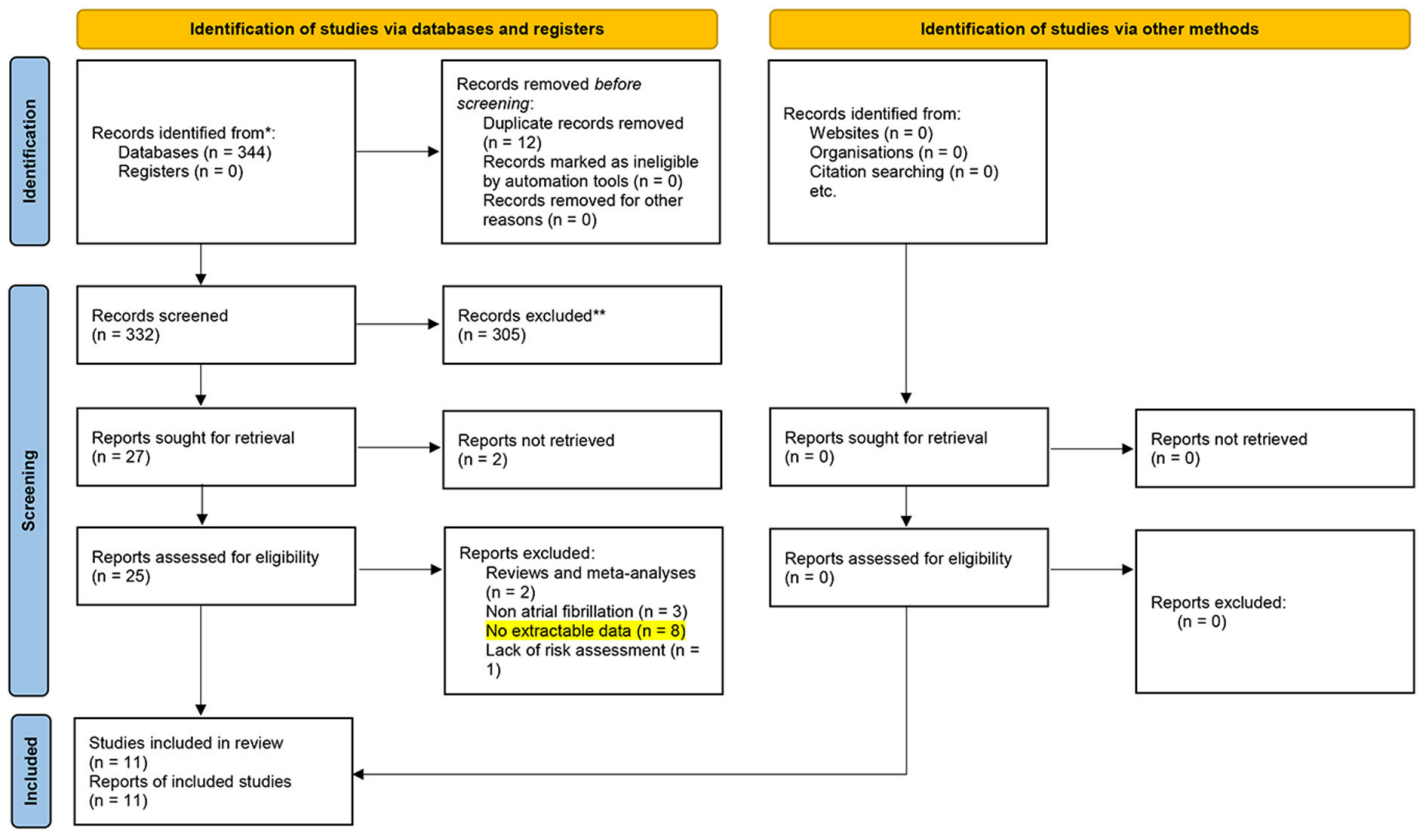
The study screening flowchart. *Consider, if feasible to do so, reporting the number of records identified from each database or register searched (rather than the total number across all databases/registers). **If automation tools were used, indicate how many records were excluded by a human and how many were excluded by automation tools.

Three hundred and forty four articles were identified from databases and manual searches. Twelve duplicate studies were initially removed, followed by title and abstract screening to exclude 305 studies, followed by full-text evaluation of the screened 27 articles. Ultimately, 11 studies were included for analysis. Figure 1 details the study screening flowchart and the reasons for excluding articles. The 11 articles included 10 case-control studies and one cohort study (7, 12–21). They originated from the three countries: Turkey (*n* = 3), China (*n* = 6), Israel (*n* = 2). In total, there were 35,121 patients with AF, of which 33,853 patients were included in the risk-related analysis because of the stroke risk grouping (941 patients were included to analyze the association of NLR levels with AF risk, and the remaining 32,912 patients were included to analyze the risk of stroke with NLR ≥3 or <3.), and the remaining 1,268 patients were included in the stroke-related analysis because of the stroke outcome grouping.

All observational studies achieved a moderate or high quality score of between 6 and 7 on the Newcastle-Ottawa Quality Assessment Form. Key study characteristics, quality assessments, patient demographics, and clinical variables of the patient population are detailed in Table 1.

**Table 1:** Study details, baseline demographic, and clinical characteristics.

**Author**	**Year**	**Country**	**Study design**	**No. of patients**	**Male gender (%)**	**Risk or outcome indicators**	NOS*
Zhao Yue et al. (12)	2018	China	Case-control studies	194	83 (43%)	CHA2DS2-VASc score	7
Murat Yalcin et al. (14)	2013	Turkey	Case-control studies	309	145 (47%)	TEE^#^	6
Sun yulei et al. (15)	2020	China	Case-control studies	100	53 (53%)	CHA2DS2-VASc score	6
Olga Perelshtein Brezinov et al. (13)	2021	Israel	Case-control studies	67	32(48%)	CHA2DS2-VASc score	6
Kahraman Cosansu et al. (7)	2017	Turkey	Case-control studies	271	117(43%)	TTR^##^	7dx
Gökhan Ertaş et al. (17)	2013	Turkey	Case-control studies	126	80 (63%)	Stroke	6
Gu Xiangting et al. (18)	2019	China	Case-control studies	190	90 (47%)	Stroke	6
Hanikzi Maimaitiyiming et al. (19)	2021	China	Case-control studies	199	114(57%)	Stroke	6
Yang Lei el al. (20)	2020	China	Case-control studies	447	253(57%)	Stroke	6
Rui el al. (21)	2015	China	Case-control studies	306	160(52%)	Stroke	6
Saliba et al. (16)	2015	Israel	Cohort study	32 912	15,932(48%)	CHA2DS2-Vasc/stroke	7

NOS^*^, the Newcastle-Ottawa Scale; TEE^#^, transesophageal echocardiography; TTR^##^, time in therapeutic range.

### NLR levels in different risk groups for AF

Among these 11 papers, six analyzed the correlation between different stroke risks and NLR levels in patients with AF, of which four used CHA2DS2-Vasc score, one used transesophageal echocardiography (TEE), and one used time in therapeutic range (TTR). One of these analyses the association between different stroke risks and different stratified NLR levels in patients with AF. The remaining five articles analyzed the association with NLR levels according to whether or not stroke occurred in AF patients.

**Figure 2:**
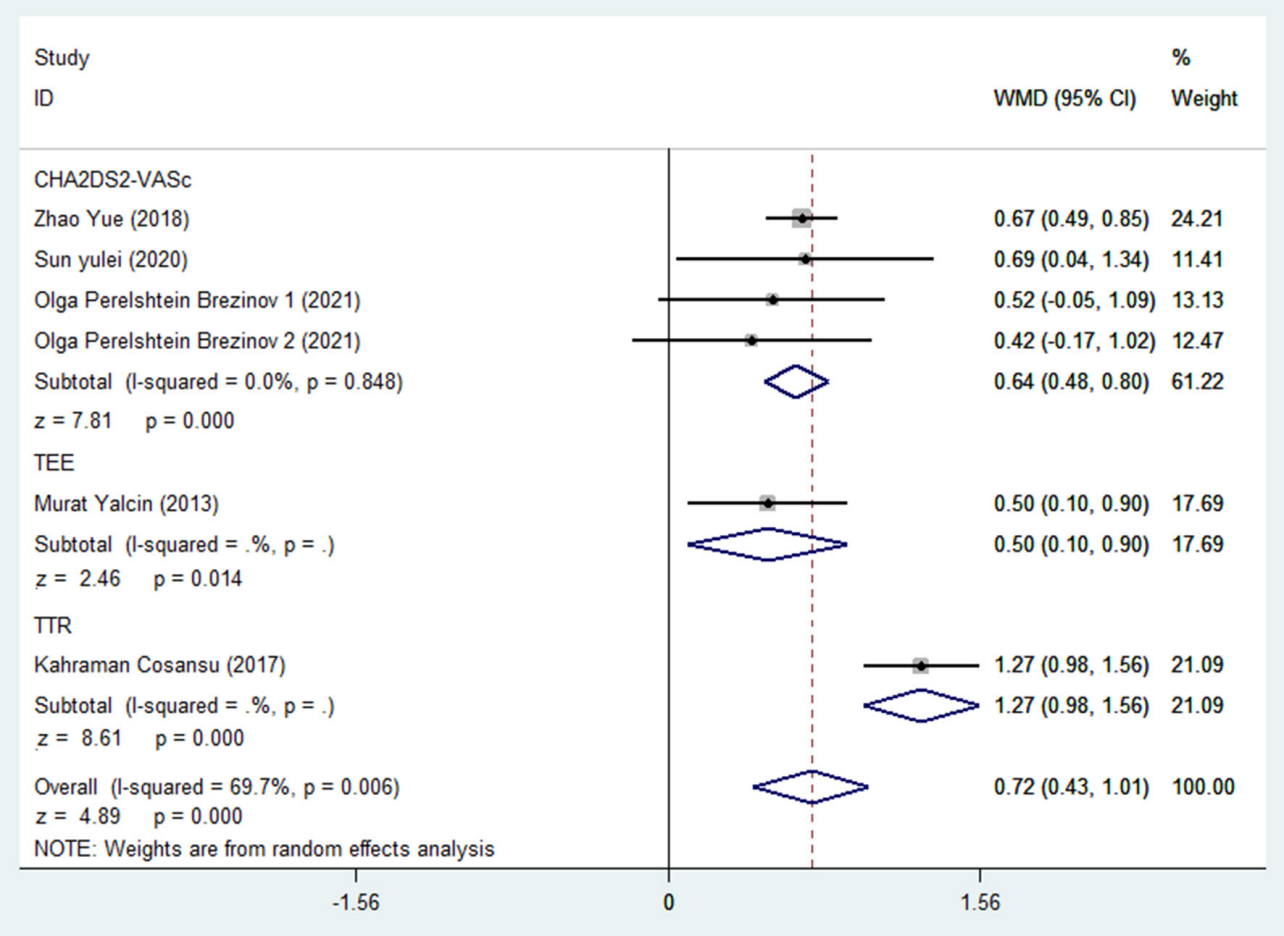
Forest plot of WMD for association between different stroke risks and NLR levels in patients with AF.

There were 941 AF patient, including 438 in the high-risk group for stroke and 503 in the low-risk group for stroke. In pooled analysis, NLR levels were significantly higher in patients with AF at higher stroke risk than in patients with AF at low stroke risk, with a WMD of 0.72 using a random-effects model (95% confidence interval: 0.43 to 1.01; *p* < 0.001, Figure 2), with a significant heterogeneity (*I*^2^ = 69.7%; *p* = 0.006). One of these studies included the NLR of normal people without AF for comparison, and we aggregated the results of this part of the data to show that the outcome indicators were basically unchanged, with a WMD of 0.64 using a random-effects model (95% confidence interval: 0.36 to 0.92), with a significant heterogeneity (*I*^2^ = 78.3%; *p* < 0.001, Supplementary Figure 1).

Further subgroup analysis showed that the heterogeneity was mainly due to different risk assessment methods. After excluding TEE and TTR, the results of the CHA2DS2-VASc score subgroup showed that the WMD obtained by using the random effect model was 0.64 (95% confidence interval: 0.48 to 0.80), with 0% heterogeneity between studies.

### NLR levels in patients with AF with or without stroke

**Figure 3:**
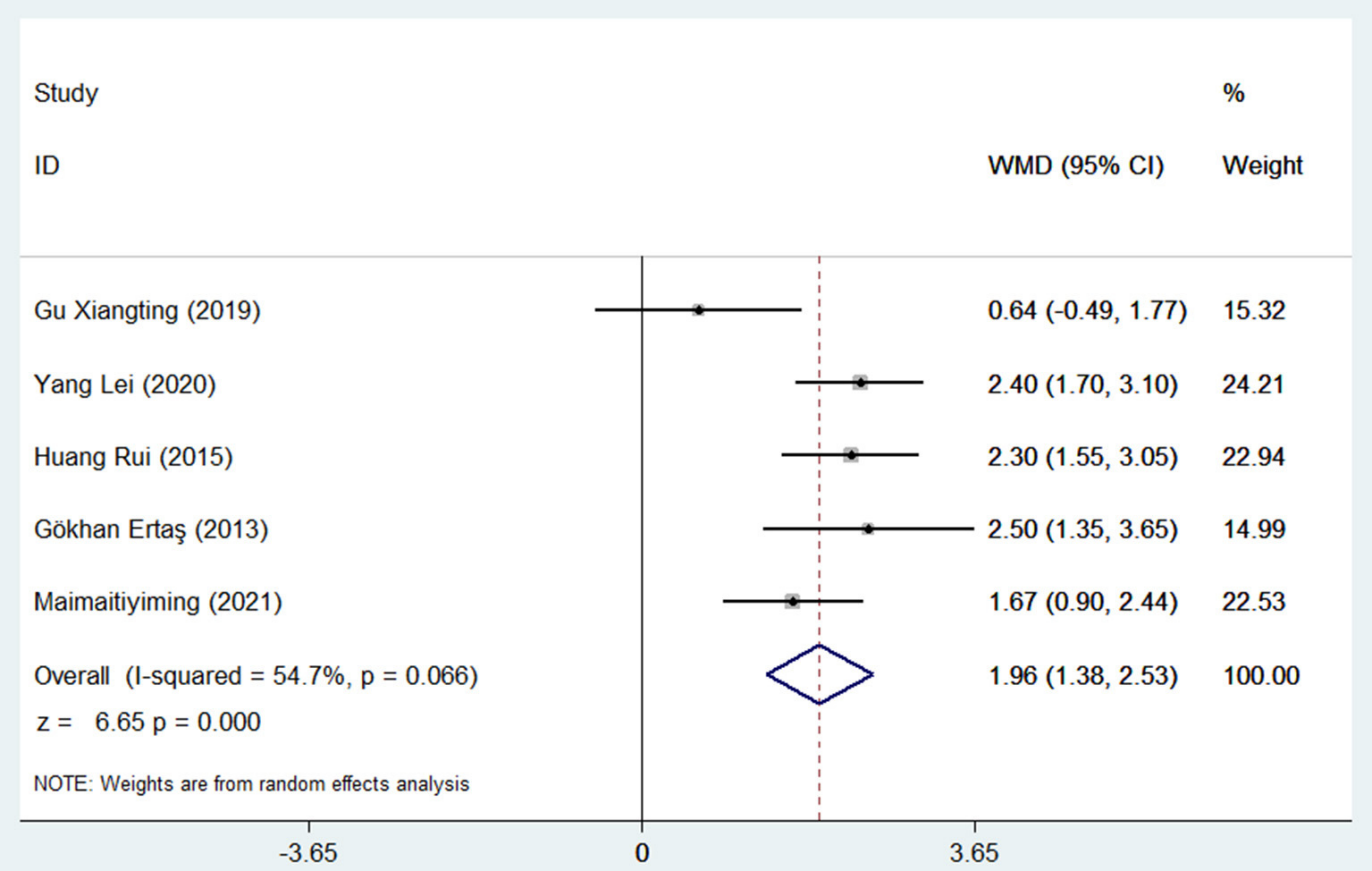
Forest plot meta-analysis of associations between different stroke outcomes and NLR levels in patients with AF.

A total of five studies with 1,268 patients included 515 strokes and 753 no strokes. After a combined analysis, the NLR levels in patients with AF who had had a stroke were significantly higher than those who had not experienced a stroke, with a WMD of 1.96 using a random-effects model (95% confidence interval: 1.38 to 2.53; *p* < 0.001, Figure 3), with a significant heterogeneity (*I*^2^ = 54.7%; *p* = 0.066).

### Association of NLR levels with stroke incidence in patients with AF

**Figure 4:**
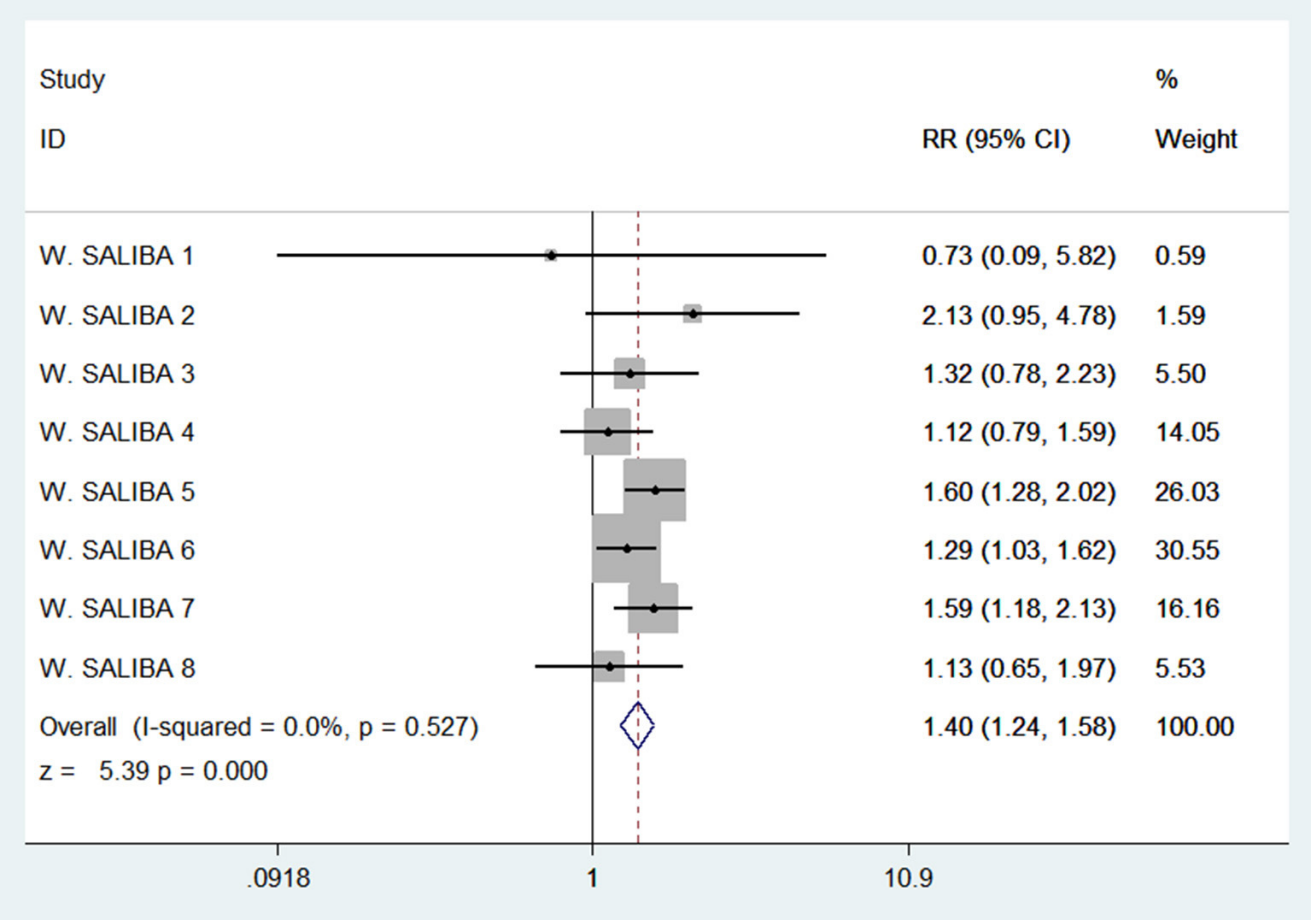
Forest plot of associations between stroke incidence and NLR levels in patients with AF.

A multi-center large sample study in Israel, the study population was divided into eight groups according to the CHA2DS2-Vasc score from 0 to 7, and each group was divided into two categories according to the NLR level ≥3 or <3. The incidence of stroke in the two kinds of NLR levels was calculated separately for each group. Our pooled analysis of stroke incidence in these eight groups by NLR level showed a 140% increased risk of stroke with an NLR≥ 3 (RR = 1.4, 95% confidence interval: 1.24 to 1.58, with 0% heterogeneity, Figure 4).

### Publication bias

There was no obvious publication bias found by Begg's Test and Egger's Test (Table 2), this suggests the absence of publication bias in the association of stroke risk with NLR levels and the association of stroke outcomes with NLR levels.

**Table 2:** Result of publication bias.

	**Begg's test**	**Egger's test**
Stroke risks and NLR^*^ levels	1.0	0.575
Stroke outcome and NLR levels	0.221	0.334

NLR

* , neutrophil-to-lymphocyte ratio.

### Sensitivity analysis

On leave-one-out analysis, the association of stroke risk with NLR levels and the results of the association of stroke outcomes with NLR levels did not change substantially. Leave-one-out analysis results are shown in Supplementary Figures 2, 3. There was no material change in magnitude for the results of replacing the effect model of the stroke incidence and NLR level (Table 3, Supplementary Figures 4, 5).

**Table 3:** Sensitivity analysis.

	**95% CI**	**z value**	***p*-value**
OR	1.42 (1.25–1.62)	5.39	0.000
RD	0.01 (0.00–0.01)	5.12	0.000

## Discussion

Patients with atrial fibrillation are at high risk of stroke and current clinical practice guidelines continue to recommend the use of the CHA2DS2-VASc score for stroke risk stratification in atrial fibrillation to identify anticoagulant candidates (22). However, actual clinical work and relevant clinical evidence have shown the limitations of the predictive value of CHA2DS2-VASc (23–27). The addition of new predictors could help improve the current approach to risk stratification.

Recently, NLR has emerged as a new potential predictor of thrombotic events that can be obtained directly from blood counts. Increased NLR levels have been reported to be associated with atherosclerotic events and as a prognostic predictor of ischemic stroke (28). High NLR levels are associated with stroke severity, adverse functional outcomes, and recurrence of ischemic events in stroke patients (29, 30). Therefore, we sought to investigate the value of NLR levels as a stratified assessment of stroke risk in patients with AF, and as a complement to the CHA2DS2-VASc score.

Through meta-analysis, we found that NLR levels were significantly higher in high-risk AF patients than in low-risk AF patients. Subgroup analysis revealed that heterogeneity stemmed from different approaches to risk assessment. The most significant difference in NLR level was in AF patients with oral vitamin K antagonists, but the INR did not reach the target, followed by B-ultrasonography to confirm the presence of atrial mural thrombus. This may be related to the adaptability of different risk assessment methods, with the CHA2DS2-VASc score being suitable for almost all patients with AF, while “INR” and “transesophageal ultrasonography” are only suitable for some AF patients (Transesophageal ultrasonography to determine if there is a thrombus). It has been reported that NLR has become an important prognostic indicator of cardiovascular disease (31). In patients with AF, elevated NLR was independently associated not only with increased risk of new-onset AF after surgery and recurrence of AF after ablation, but also with the presence of LA thrombus and increased risk of stroke in patients with AF (14, 16, 32). This may be the reason why inflammation is closely related to the pathological mechanism of AF and the mechanism of mural thrombosis. In addition, we attempted to include the normal population in the low stroke risk group for comparison. The NLR level in the high stroke risk group was still significantly higher than that in the low risk group, and the results were stable, with no significant changes in WMD values. Therefore, NLR may serve as a potential predictor of stroke risk in patients with AF.

Current guidelines classify AF into subtypes by pattern, paroxysmal AF (PAF), persistent AF, long- standing persistent AF, and permanent AF (33). Studies suggest that different subtypes of AF have different levels of stroke risk (34, 35). Despite the CHA2DS2-VASc score has high sensitivity for stroke risk, its specificity is low, with a C-statistic of 0.67 for predicting thromboembolic outcome (36). Based on the above, we searched and screened the literature and performed a systematic evaluation to analyze the correlation between different subtypes of AF and NLR levels (Supplementary Figure 6 and Supplementary Table 1). The results revealed that patients with AF had significantly higher NLR levels than those without AF, with a WMD value of 0.57 (95% CI = 0.37–0.78, *p* < 0.001). In the subgroup analysis, the NLR levels of patients with non-paroxysmal AF were not statistically different from those of the AF-free population, with a WMD value of 0.19 (95% CI = −0.16–0.53, *p* = 0.295), unlike the NLR levels of patients with paroxysmal AF, which were significantly higher than those of the AF-free population, with a WMD value of 0.76 (95% CI = 0.42–1.11, *p* < 0.001). We suggest that NLR levels may be more clinically relevant for patients with PAF as a complementary stratification marker for AF stroke risk stratification or CHA2DS2-VASc score.

Further analysis of NLR levels in patients with AF who had a stroke versus those who did not, we found higher levels of NLR in AF patients who had a stroke. This suggests that high NLR levels are strongly associated with stroke in patients with AF. On a case-by-case basis, the heterogeneity was mainly derived from one study, and the possible cause of the heterogeneity could not ultimately be determined because the original data were not available. After the study was excluded, the results remained stable. NLR levels are closely associated with stroke in AF patients.

We performed a meta-analysis of data from a large multicenter Israeli study that divided patients with AF into two groups by NLR levels of ≥3 and <3 and found that the risk of stroke was 1.4 times higher in patients with AF with NLR ≥3 than with NLR <3. In this study, among the AF population with NLR ≥3, 19,726 (83.2%) had a CHA2DS2-VASc score ≥2, and 3,986 (16.8%) had a score <2. Among the AF population with NLR <3, 8502 (92.4%) had a CHA2DS2-VASc score ≥2, and 698 (7.6%) had a score <2. The study conclusively showed that NLR is directly associated with stroke risk in AF, and together with CHA2DS2-VASc score, it can also help to stratify stroke risk in patients with low CHA2DS2-VASc scores, allowing patients to receive timely treatment and reduce stroke risk.

Inflammation has been found to be an important pathological mechanism involved in the occurrence, development and prognosis of stroke. Systemic inflammatory response index (SIRI) can be used as a predictor of futile endovascular reperfusion, and higher SIRI levels are associated with an increased risk of adverse outcomes in stroke patients at 3 months after vascular recanalization (37). In stroke patients receiving intravascular therapy, higher NLR values indicate a higher risk of early neurological deterioration, and can also predict the risk of symptomatic hemorrhagic transformation after vascular recanalization (38, 39). The possible mechanism is that a higher level of NLR is closely related to platelet activation and thrombosis. On the one hand, neutrophils participate in the formation of atherosclerosis by secreting various inflammatory mediators, and aggravate endothelial cell dysfunction (40, 41), which may be related to neutrophils increasing the release of proteolytic enzymes or free oxygen free radicals and other inflammatory mediators (42). On the other hand, higher levels of lymphocytes can upregulate the anti-inflammatory cytokine interleukin (IL)-10 and inhibit inflammatory cytokines including tumor necrosis factor-α and IL-6, thereby exerting anti-inflammatory effects (43). In addition, it has been experimentally demonstrated that regulatory T cells and B cells have regulatory functions to reduce ischemic tissue volume and improve neurological deficits during ischemic stroke (44). NLR is the ratio between cells that mediate two different immune pathways (45). In conclusion, NLR is a potential predictive biomarker of stroke risk in AF patients and may serve as an important complementary risk stratifier to the CHA2DS2-VASc score.

This study also has some limitations. Firstly, NLR reflects the overall inflammatory status of the body. If the patient has mild urinary tract infection or chronic pulmonary interstitial disease without obvious symptoms and signs, the reference value of NLR may be affected. Therefore, establishing a reference standard for NLR levels of chronic inflammation, excluding the interference of other inflammations, and correcting the NLR value may further improve the value of NLR for stroke risk stratification. Secondly, due to the difficulty in obtaining the original data, this study lacks a separate analysis of patients with CHA2DS2-VASc score <2. Therefore, more relevant research is needed to further update the future research and increase the persuasiveness of NLR as a stroke risk assessment for patients with low CHA2DS2-VASc score. Thirdly, the RR value of this meta-analysis is small, which needs to be treated with caution by clinicians. It is recommended to use NLR on the basis of CHA2DS2-VASc score, and it needs to be evaluated in combination with the patient's own situation. Finally, as described in a previous meta-analysis on NLR, the cutoff values for NLR was not determined (46). The NLR cutoff values of each article in the studies on the relationship between stroke risk and NLR level in patients with AF included in this meta-analysis was different. Different NLR cutoff values may affect the accuracy of the analysis. Therefore, more relevant studies are needed to establish standard NLR cutoff values.

## Conclusion

In summary, our study suggests that high NLR values are associated with a high risk of stroke in AF patients. The incidence of stroke in AF patients with NLR ≥3 was 1.4 times higher than that with NLR <3. NLR can be used as a supplemental risk assessment for CHA2DS2-VASc score, especially for AF patients with CHA2DS2-VASc score <2. In the future, more relevant studies are needed to establish the correction formula of NLR and the standard cut-off value of NLR, which can be better applied to clinical prediction of stroke risk in AF.

## Data availability statement

The original contributions presented in the study are included in the article/Supplementary material, further inquiries can be directed to the corresponding author.

## Author contributions

BH and ML conceived and wrote the manuscript together. RL, YZ, and XH performed literature search, screening, and data collection. All authors contributed to the article and approved the submitted version.
