# Supplementary material for: Predictive value of neutrophil to lymphocyte ratio for ischemic stroke in patients with atrial fibrillation: A meta-analysis
Source: Front Neurol. 2022 Dec 12;13:1029010. doi: 10.3389/fneur.2022.1029010 (PMC9792176; doi:10.3389/fneur.2022.1029010)
Supplement: Supplementary file 1 [file Data_Sheet_1.PDF]

Supplementary Material

1 Supplementary Figures

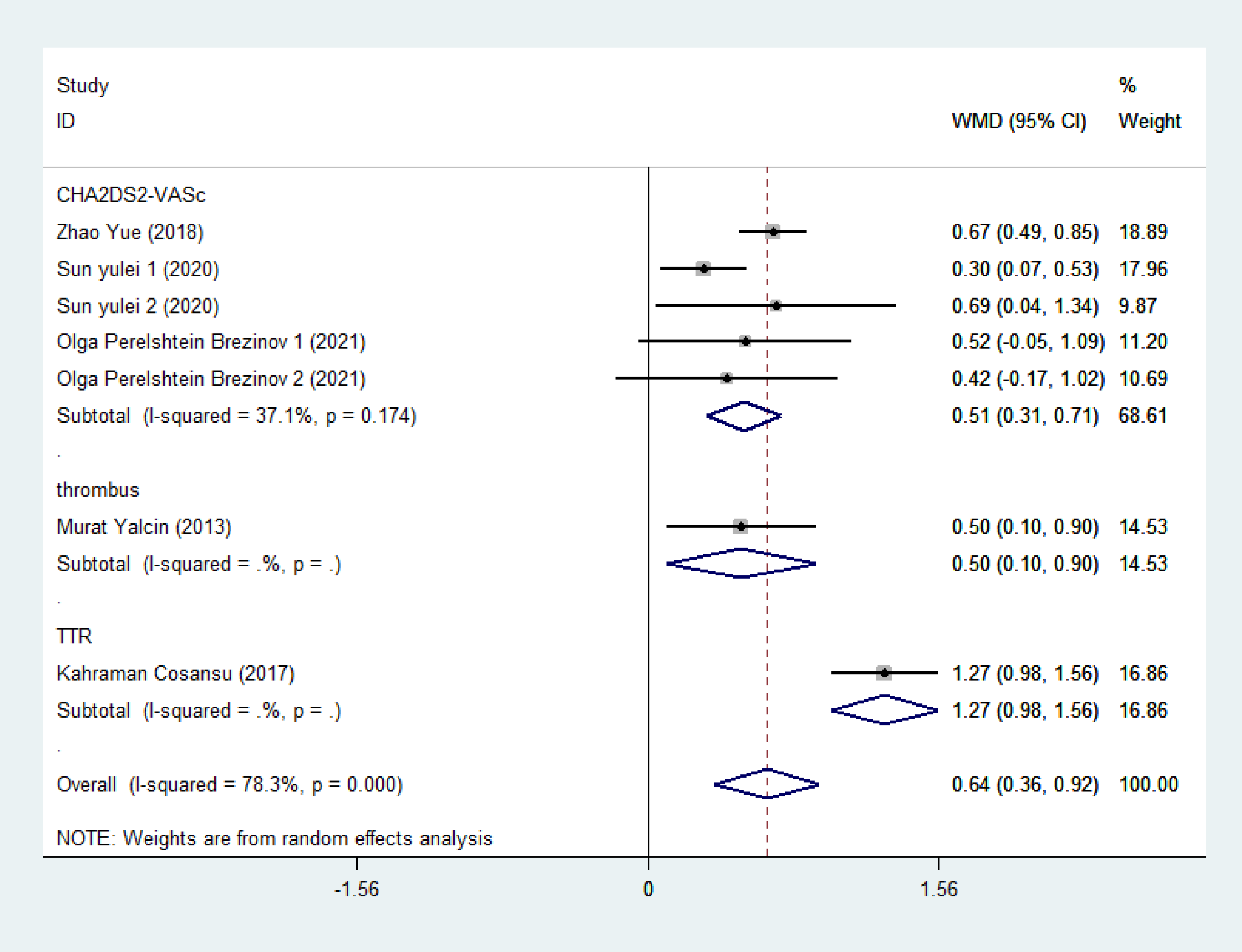

Supplementary Figure 1. One of these studies included the NLR of normal people without AF for comparison, and we aggregated the results of this part of the data to show that the outcome indicators

were basically unchanged, with a WMD of 0.64 using a random-effects model (95% confidence interval: 0.36 to 0.92), with a significant heterogeneity (I^2 =78.3%; p<0.001).

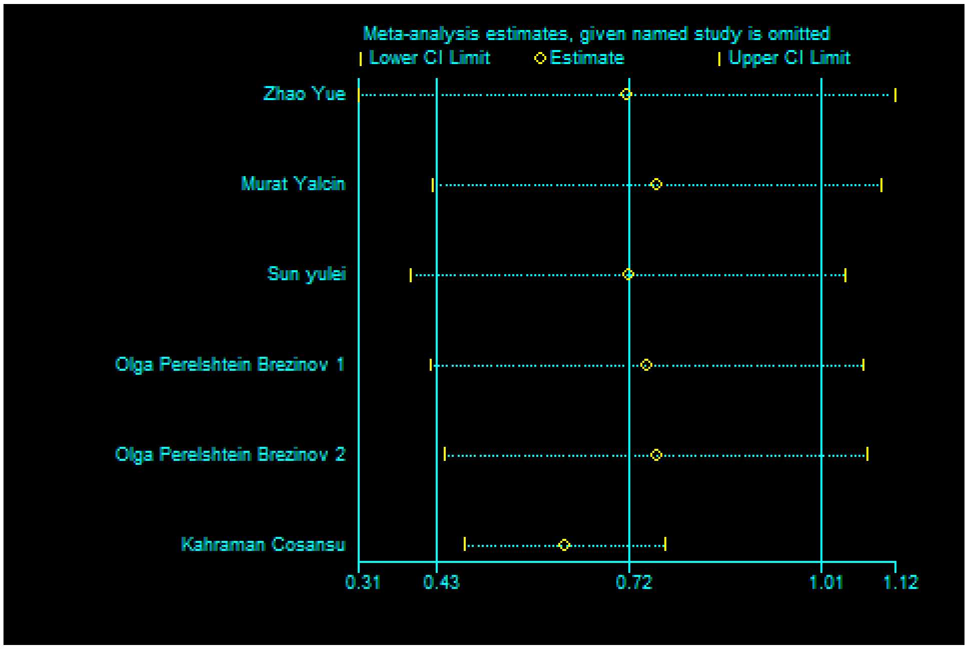

Supplementary Figure 2. Leave-one-out analysis of the association between stroke risk and NLR levels.

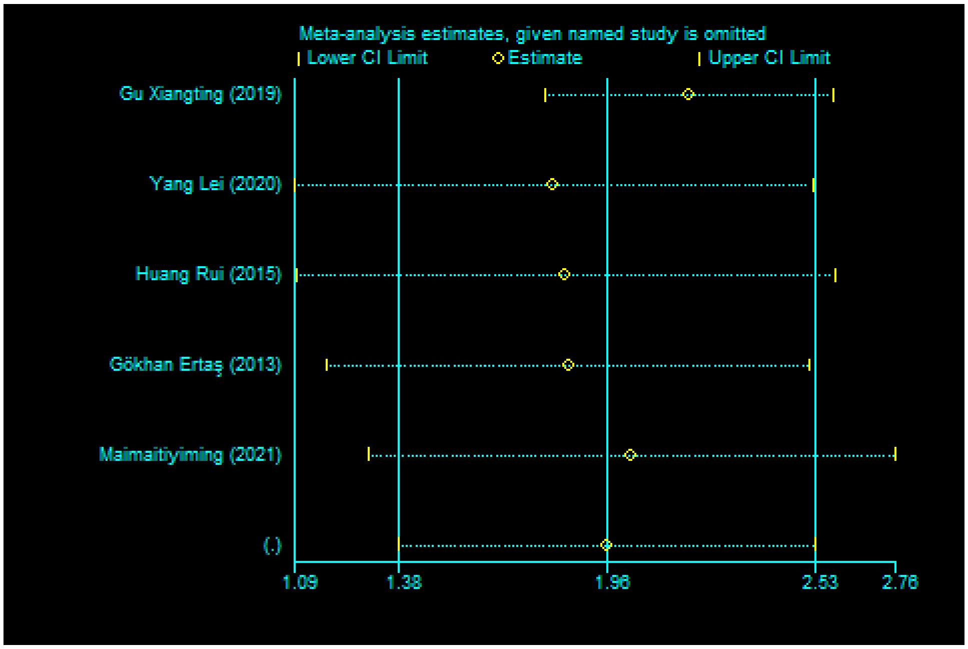

Supplementary Figure 3. Leave-one-out analysis of the association between the results of the association of stroke outcomes and NLR levels.

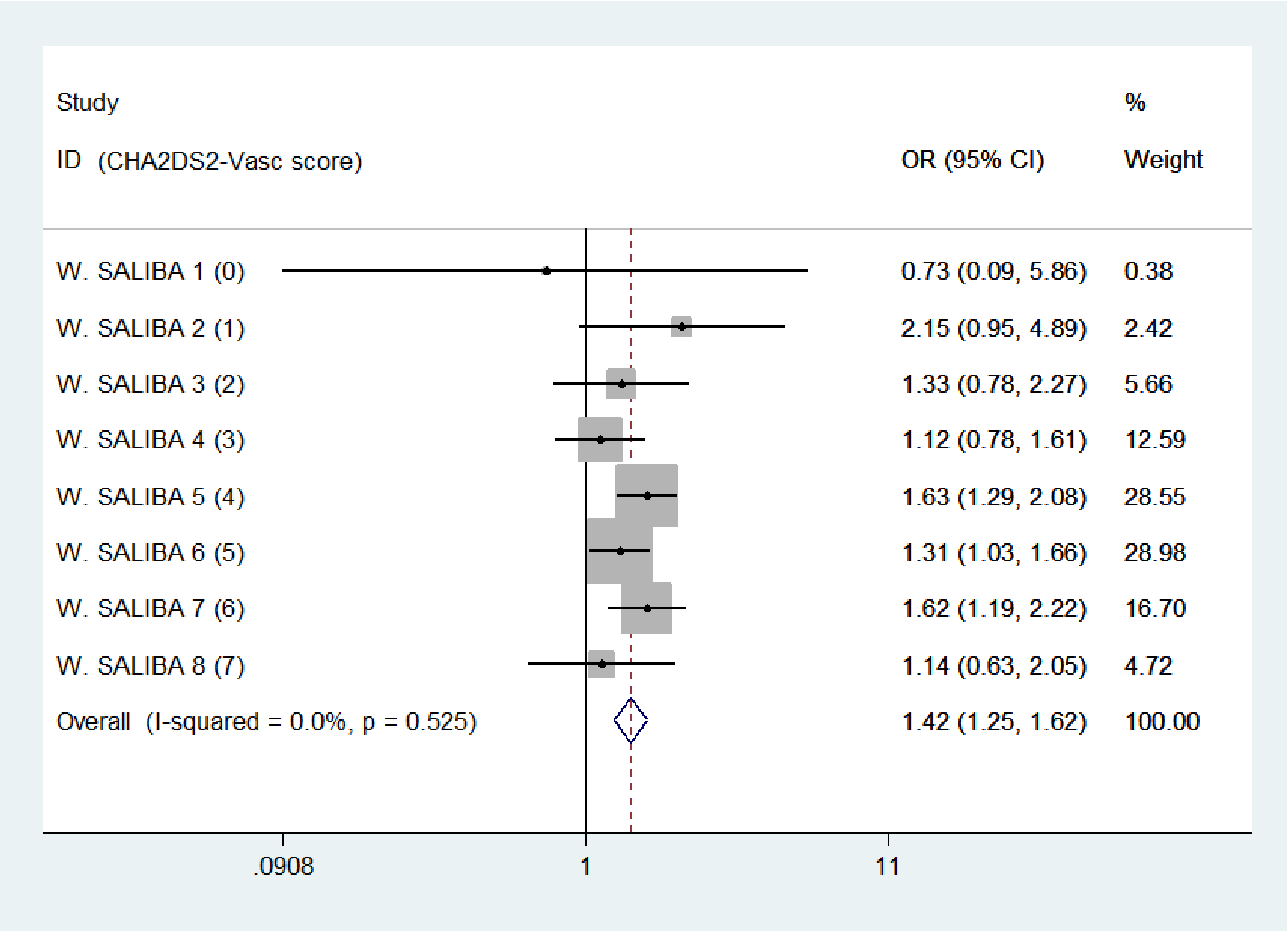

Supplementary Figure 4. Forest plot of associations between stroke incidence and NLR levels in patients with AF

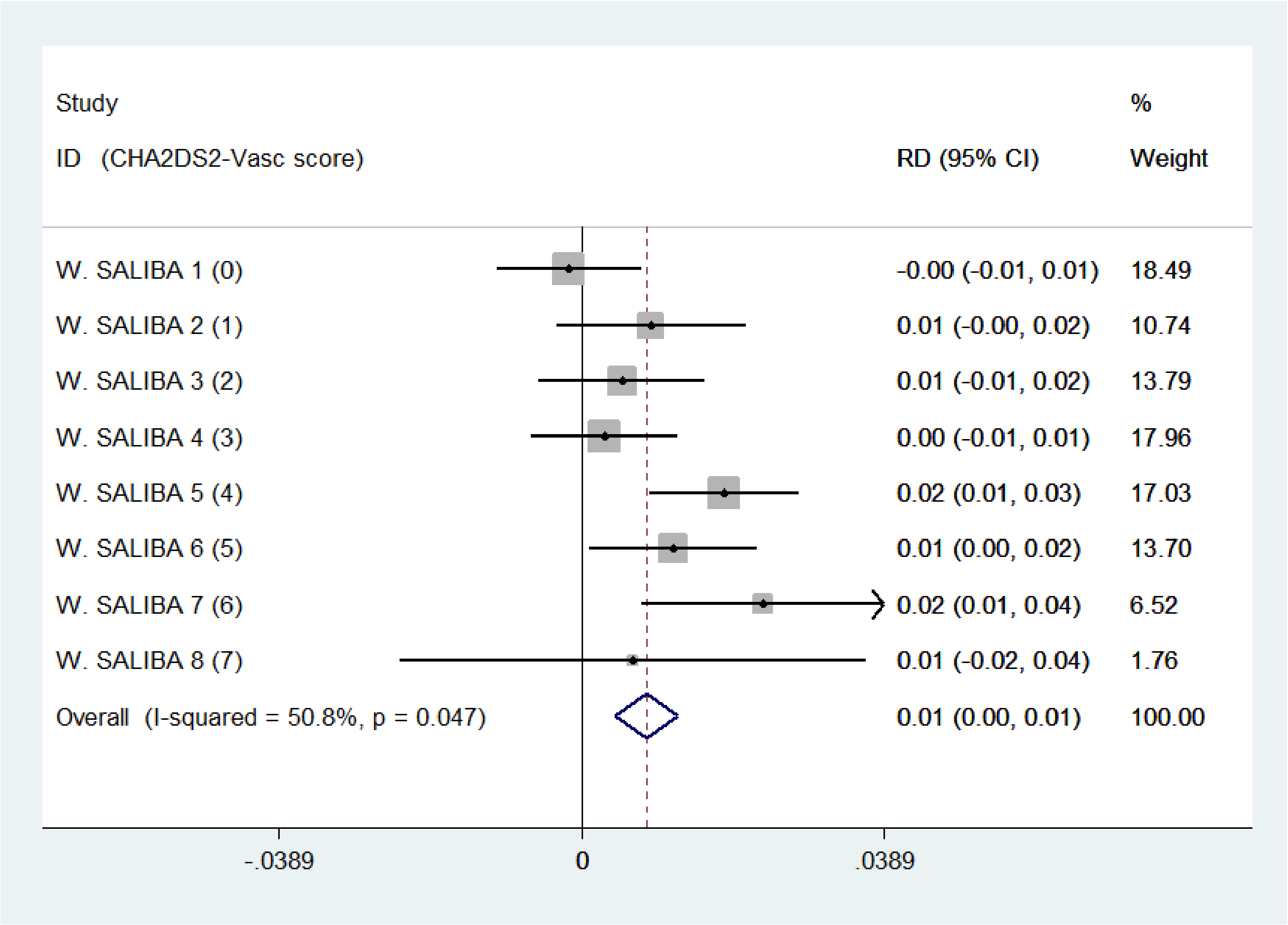

Supplementary Figure 5. Forest plot of associations between stroke incidence and NLR levels in patients with AF

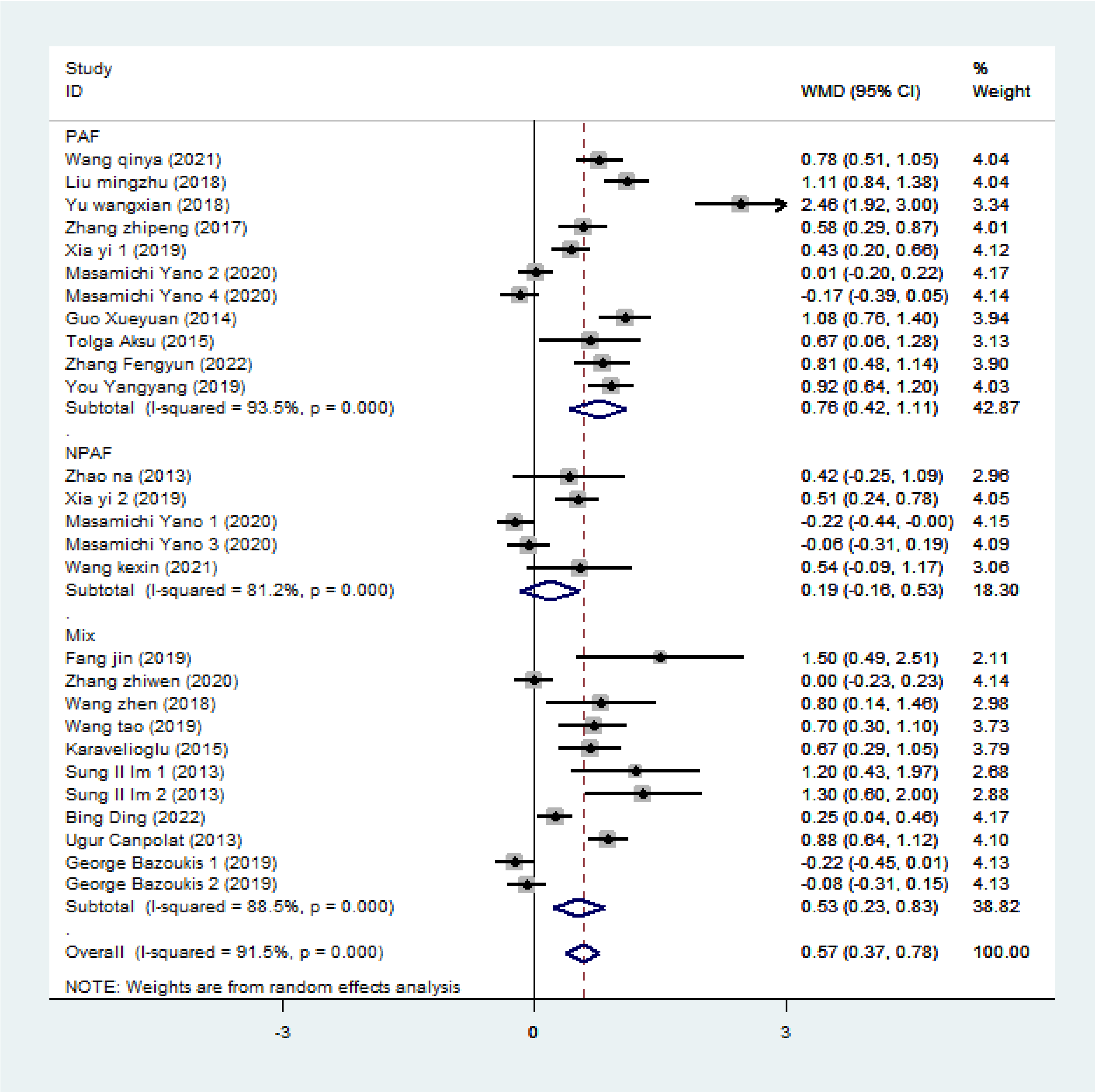

NPAF, Non-paroxysmal atrial fibrillation. Mix, including PAF and NPAF.

Supplementary Figure 6. Forest plot of WMD for association between NLR levels and AF

Supplementary Table 1 Characteristics of the articles included.

Author	Year	type of atrial fibrillation	Country	Patients (recurrence)	Follow-up	NOS
Wang qinya[1]	2021	PAF	China	186(38)	Within 12 months	5
Liu minxingzhu[2]	2018	PAF	China	89(27)	Within 12 months	5
Zhang zhiwen[3]	2020	Mix	China	166(48)	More than 6 months	7

Xia yi [4]	2019	Mix	China	554(138)	More than 3 months	5
Wang tao[5]	2019	Mix	China	129(40)	More than 3 months	5
Masamichi Yano[6]	2020	Mix	Japan	633(203/162)	Within 3 months /More than 3 months	5
Bing Ding[7]	2022	Mix	China	263(70)	More than 3 months	8
George Bazoukis [8]	2019	Mix	Greece	346(97/80)	Within 3 months /More than 3 months	7
Wang kexin[9]	2021	PerAF	China	91(37)	More than 3 months	5
Tolga Aksu[10]	2015	PAF	Turkey	49(7)	More than 3 months	5

Supplementary Table 1. A total of 2506 patients (10 articles) were included. Table 2 shows the characteristics of the included articles. All studies were performed with radiofrequency ablation (RA) for cardioversion, including 7 from China, 1 from Japan, 1 from Turkey and 1 from Greece. The follow-up time was from 1 month to 12 months after cardioversion. The NOS scores were all above 5 points, 1 paper was 8 points, and 2 papers were 7 points.

[1] W. Qingya, L. Jia, Z. Yuzhen, et al. The predictive value of multi-index combined evaluation model for recurrence of paoxysmal atrial fibrillation after radiofrequency catheter ablation [J]. journal of Clinical Cardiology, 2021, 37(1): 7.

[2] L. Mingzhu,Z. Lijun. Effect of peripheral blood neutrophil to lymphocyte ratio and high-sensitivity C-reactive protein on recurrence in patients with paroxysmal atrial fibrillation after radiofrequency ablation [J]. Clinical Medicine of China, 2018, 34(5): 4.

[3] Z. Zhiwen, L. Changbo, W. Ting, et al. Clinical significance of neutrophil-lymphocyte ratio for the recurrence of radiofrequency ablation in patients with fibrillation [J]. Chinese Journal of Cardiovascular Research, 2020, 18(08): 729-733.

[4] X. Yi, Y. Yiheng, L. Dekai, et al. Comparison of predictive value of NLR for recurrence after radiofrequency ablation of paroxysmaland persistent atrial fibrillation [J]. Chin J Evid Based Cardiovasc Med, 2019, 11(8): 4.

[5] W. Tao, Predictive value of preoperative neutrophil-lymphocyte ratio in recurrence of atrial fibrillation after radiofrequency ablation, Soochow university, 2019.

[6] M. Yano, Y. Egami, K. Ukita, et al. Atrial fibrillation type modulates the clinical predictive value of neutrophil-to-lymphocyte ratio for atrial fibrillation recurrence after catheter ablation [J]. International journal of cardiology Heart & vasculature, 2020, 31: 100664.

[7] B. Ding, P. Liu, F. Zhang, et al. Predicting Values of Neutrophil-to-Lymphocyte Ratio (NLR), High-Sensitivity C-Reactive Protein (hs-CRP), and Left Atrial Diameter (LAD) in Patients with Nonvalvular Atrial Fibrillation Recurrence After Radiofrequency Ablation [J]. Medical Science Monitor: International Medical Journal of Experimental and Clinical Research, 2022, 28: e934569-934561.

[8]G. Bazoukis, K. Letsas, K. Vlachos, et al. Simple hematological predictors of AF recurrence in patients undergoing atrial fibrillation ablation [J]. Journal of geriatric cardiology : JGC, 2019, 16(9): 671-675.

[9] W. Kexin, The risk factors of long-standing persistent atrial fibrillation after radiofrequency catheter ablation, Dalian Medical University, 2021.

[10] T. Aksu, E. Baysal, T.E. Guler, et al. Predictors of atrial fibrillation recurrence after cryoballoon ablation [J]. Journal of Blood Medicine, 2015, 6: 211.
